# Online game player churn prediction based on multiplex social influence through network embedding

**DOI:** 10.1371/journal.pone.0356200

**Published:** 2026-08-20

**Authors:** Ming Guo, Yan Liu

**Affiliations:** 1 School of Management, Zhejiang University of Science and Technology, Hangzhou, China; 2 Business School, NingboTech University, Ningbo, China; Institute of Management Sciences Peshawar, PAKISTAN

## Abstract

Accurately predicting player churn is crucial for designing effective retention strategies and driving revenue in the online gaming industry. While existing predictive models achieve reasonable performance relying on individual demographic and behavioral data, they largely overlook the multiplex social influence arising from diverse, overlapping player relationships. To address this gap, we propose a novel Multiplex Social Influence (MSI) framework for player churn prediction. Specifically, we construct multiplex social networks encompassing both explicit friendship ties and implicit Player-versus-Environment (PvE) teammate relationships. We then apply a network embedding algorithm called MLNRL to efficiently extract multi-scale structural information from these distinct networks. These embeddings are concatenated to form a unified multiplex social influence representation and combined with players’ behavioral attributes for downstream classification. Extensive experiments on a real-world online game dataset validate the superiority of our proposed approach. Quantitatively, the MSI framework paired with an SVM (RBF kernel) classifier achieves outstanding prediction performance (accuracy: 0.93, F1-score: 0.93, AUC: 0.98. Furthermore, rigorous ablation studies, comprehensive robustness tests (covering network embedding algorithms, fusion strategies, and data imbalance issues), and parameter sensitivity analyses collectively confirm that integrating multiplex social influence yields robust synergistic effects, significantly outperforming baseline models built on single relationship types. These findings definitively confirm the effectiveness and necessity of incorporating multiplex social structures into data-driven churn prediction models for online game services.

## 1 Introduction

Digital games have become an indispensable component of contemporary entertainment. In 2025, the number of game players worldwide was 3.6 billion, with total industry revenue reaching approximately 188.8 billion U.S. dollars [1]. An online game is a form of multiplayer digital game that enables player interactions via network connectivity and accounts for the largest share of the global game market in terms of both player count and financial revenue [2]. In recent years, the proliferation in network technologies and mobile devices has significantly driven the expansion of the online game industry. Valued at approximately USD 144.6 billion in 2025, the global online game market is projected to reach USD 280.3 billion by 2033, reflecting a compound annual growth rate (CAGR) of 8% from 2026 to 2033 [3].

Unlike console games and PC single games that typically rely on one-time sales, online games predominantly employ freemium or free-to-play business model. Under these models, online games are typically accessible at no initial cost, with players having the option to purchase in-game items to enhance their gameplay experience. Consequently, the revenue is primarily generated through in-game microtransactions with a massive player community [4]. The freemium or free-to-play business model also leads to a non-contractual relationship between players and game providers, allowing players to disengage with few constraints [5]. As a result, player retention has become a critical determinant of profitability for online game companies, thereby establishing player churn prediction as a significant research topic. Player churn prediction aims to identify whether—and in some cases when—players are likely to discontinue gameplay in the future [6]. Accurate player churn prediction is crucial for understanding player loyalty and improving retention rates, enabling game companies to implement timely and targeted interventions to incentivize at-risk players to remain engaged.

To date, a substantial body of research has focused on player churn prediction, with most studies relying on supervised machine learning models and reporting robust prediction performance [7]. These approaches typically leverage players’ demographic attributes and in-game behavioral features as inputs for prediction [6]. However, they largely neglect the relationship information embedded in player interactions. In online games, players often engage in multiple types of relationships, such as friendship ties and PvE teammate relationships; these heterogeneous interaction types are collectively referred to as multiplex relationships [8]. Such multiplex relationships can significantly shape players’ engagement and decision-making processes and, consequently, influence their churn behavior. Prior studies have demonstrated that multiplex relationship information can affect individual behavior through multiplex social influence in various domains, including human resource management, marketing, and investment [9]. Therefore, beyond players’ demographic attributes and in-game behavioral features, incorporating multiplex social influence represents a promising avenue for advancing the performance of player churn prediction models.

This raises the central question of how to effectively quantify the multiplex social influence experienced by each player in online games. While social influence is intrinsically tied to network topology, conventional approaches—such as classical graph-theoretic metrics [10] (e.g., centrality measures and clustering coefficients)—often produce representations with limited structural fidelity or incur high computational costs. Degree centrality captures only first-order interactions and fails to account for the indirect influence propagated through the network, whereas betweenness centrality entails cubic time complexity with respect to the number of nodes, rendering it intractable for large-scale networks [11]. In contrast, network embedding or network representation learning (NRL) methods project nodes into a low-dimensional latent space while preserving multi-scale nonlinear structural properties or high-order structural proximities of networks. Such embeddings can be efficiently learned using scalable network embedding algorithms and effectively capture both direct and indirect social influence [12,13]. Network embeddings provide a suitable and scalable proxy for multiplex social influence in online game networks.

In this paper, we propose a novel player churn prediction framework that utilizes multiplex social influence via advanced network embedding to significantly improve prediction accuracy in online games. Specifically, our approach is structured as follows: First, we construct multiplex social graphs based on two distinct interaction channels—explicit friendship ties and implicit PvE teammate relationships. Second, we employ a highly scalable, multi-scale network representation learning algorithm (MLNRL) to generate structural embeddings for each respective network. By avoiding explicit matrix computations, this algorithm efficiently preserves hierarchical graph properties. These distinct embeddings are subsequently concatenated to form a unified, comprehensive representation of the multiplex social influence experienced by each player. Third, the learned social embeddings are fused with individual demographic attributes and in-game behavioral features, providing a rich, joint feature space for supervised machine learning classifiers to predict churn. Finally, extensive experiments on a large-scale, real-world online game dataset demonstrate the effectiveness and superiority of our framework. Empirical results confirm that integrating heterogeneous social ties yields a significant synergistic advantage over traditional, single-layer relationship models.

This study makes three key contributions to the literature on player behavior mining and social network analysis. First, we propose a novel player churn prediction framework driven by multiplex social influence. By jointly modeling explicit friendship ties and implicit PvE teammate relationships, we demonstrate that integrating heterogeneous social networks yields a significant synergistic effect, overcoming the limitations of relying on isolated, single-layer relationships. Second, from a methodological perspective, we highlight the critical role of multi-scale network structural information. We demonstrate that employing an advanced network embedding algorithm (MLNRL)—which implicitly factorizes the SPPMI matrix in linear time and utilizes an adversarial cascade—can preserve hierarchical social structures in a highly efficient manner while preventing over-smoothing, thus setting a new standard for scalability and structural expressiveness in gaming networks. Finally, we provide robust empirical evidence and actionable value for the gaming industry. Through rigorous robustness checks and ablation studies, our framework consistently achieves exceptional prediction performance. Notably, its ability to maintain both high precision and high recall empowers game operators to accurately target high-risk churners for retention campaigns, minimizing false alarms and optimizing operational resources.

The remainder of this paper is organized as follows. Section 2 reviews related work on online games, player churn prediction, influence of multiplex relationships and multiplex social influence, and network embedding. Section 3 details the proposed player churn prediction framework. Section 4 presents the experimental setup, comparative analyses, and results. Finally, section 5 summarizes the conclusions, contributions, and limitations of this study.

## 2 Literature review

### 2.1 Online games

Online games are computer or mobile games in which multiple players participate simultaneously through a network. A key distinction from traditional offline games is their capacity to support sustained player-to-player interaction in addition to player-to-environment engagement. Massively multiplayer online role-playing games (MMORPGs) represent a prominent category of online games, characterized by their ability to host thousands of players concurrently. The rich social interaction features and continuous content updates in MMORPGs contribute significantly to their sustained appeal to players [14].

Unlike console games and PC single-player games, which primarily generate revenue through one-time purchases, most online games adopt the freemium model. Under this pricing strategy, the core product or service is offered free of charge, while revenue is generated through additional items or value-added services [15]. In other words, players can access online games for free and may purchase optional in-game items to improve their gameplay experience or strengthen their characters. The freemium model offers several advantages. First, by lowering entry barriers, it promotes widespread adoption, enabling a larger pool of players to try the game and creating opportunities for upselling at a later stage [16]. Second, it facilitates network effects, whereby the overall value and attractiveness of the game increase as the player base expands [17]. However, the freemium model also entails notable drawbacks. By creating a non-contractual relationship between players and game providers, it allows players to disengage at any time without constraints [18]. Moreover, while online game companies typically emphasize user acquisition through marketing efforts in the early stages of a game’s lifecycle, attention gradually shifts toward player retention as growth slows. Importantly, even small changes in retention can yield a disproportionately large impact on profits—for example, a 5% increase in customer retention can lead to approximately a fourfold increase in revenue [19]. Consequently, maintaining or enhancing player retention has become one of the most critical concerns for online game companies.

A central challenge in the online gaming industry is predicting player attrition, that is, identifying whether a player is likely to discontinue participation. To address this challenge, player churn prediction has emerged as a critical analytical tool. Prior research indicates that players who exhibit behavioral patterns diverging systematically from those of retained players are prone to churn [20]. By accurately identifying these at-risk individuals in advance, game companies can implement proactive, timely, and targeted interventions—such as personalized incentives or tailored content recommendations—to sustain engagement and mitigate churn.

### 2.2 Player churn prediction

Churn prediction is a critical challenge across a wide range of industries, including telecommunications, finance, customer relationship management (CRM), and human resource management [21–26]. For example, Ly and Son [25] point out that customer churn can lead to a significant drop in profits for telecommunication companies as customers always have a variety of choices and tend to choose the companies that can offer them better quality but less expensive services, while the cost of acquiring new customers for e-commerce platforms is often higher than the cost of retaining existing customers [27]. In parallel with these cross-sector applications, a substantial and growing body of research has focused specifically on the gaming industry. In recent years, scholars have increasingly explored diverse modeling techniques to enhance the accuracy and robustness of player churn prediction [28,29]. Significant progress has been made in this area, with the majority of studies concentrating on whether a player will discontinue gameplay. Most existing approaches frame churn prediction as a binary classification problem and employ supervised machine learning models such as logistic regression (LR), support vector machine (SVM), random forest (RF), and other traditional classifiers [30]. For example, Runge et al. [5] applied LR and SVMs to predict churn behavior in two casual social games, while Kiguchi et al. [31] utilized decision tree and random forest to model player churn in a Japanese role-playing game. Beyond conventional machine learning methods, researchers further demonstrated the effectiveness of deep learning approaches—such as convolutional neural networks (CNNs) and Long Short-Term Memory (LSTM)—in capturing complex temporal and behavioral patterns inherent in player churn [32,33].

However, existing approaches share a common limitation: they predominantly rely on only two categories of features for churn prediction, namely players’ demographic attributes and in-game behavioral features. For instance, Perianez et al. [6] categorized predictive features into four dimensions: player attention, player loyalty, player intensity, and player level. Player attention captures engagement-related indicators such as daily playtime and registration duration; player loyalty reflects long-term commitment through metrics such as active days and payment days; player intensity describes the frequency of in-game activities; and player level represents character level or progression. Similarly, Bertens et al. [34] employed playtime-based and level-based features for churn prediction. Beyond these predefined attributes and behavioral features, some studies have further leveraged feature engineering techniques to derive more expressive behavioral features from gameplay logs to enhance prediction performance.

Nevertheless, these approaches have largely overlooked the relationship information among players, despite evidence suggesting that player relationships can significantly influence churn behavior [35]. As summarized in Table 1, the majority of prior studies focus primarily on players’ demographic attributes and behavioral features, giving limited consideration to social interactions and relationships. Although several studies have begun to incorporate player relationship information, they typically rely on classical graph-theoretic metrics, such as degree centrality, as predictive features. For instance, Óskarsdóttir et al. [10] extracted features from explicit friendship networks—specifically, the proportion of churners among a player’s friends—demonstrating that incorporating such degree-based information significantly enhances churn prediction accuracy. However, such features provide only partial structural insights and fail to capture higher-order or multiplex relationship patterns. Building upon the concept of complex relationships, recent state-of-the-art studies have begun to explore multiplex network architectures. For example, Han et al. [36] utilized Graph Convolutional Networks (GCNs) to capture user interactions across multiple social graphs, encompassing guild affiliations and in-game trading behaviors. Despite these advancements, the existing literature predominantly focuses on either isolated friendship networks or macro-level socio-economic interactions (e.g., guild memberships and virtual trades). A critical research gap remains regarding the synergistic interplay of micro-level gaming behaviors. To the best of our knowledge, no prior research has investigated the multiplex social influence formed by the specific combination of explicit friendship ties and implicit PvE teammate relationships. Consequently, the rich social structures inherent in online gaming networks remain insufficiently exploited in current player churn prediction models.

**Table 1:** Different types of features used by player churn prediction models.

Literature	Individual Features (Attributes & Behavior)	Social / Relational Information	Relational Representation
Hadiji et al. [4]	√	×	×
Runge et al. [5]	√	×	×
Kim et al. [32]	√	×	×
Perišić and Pahor [37]	√	×	×
Kiguchi et al. [31]	√	×	×
Xiong et al. [38]	√	×	×
Zhao et al. [2]	√	×	×
Guo et al. [39]	√	×	×
Wang et al. [40]	√	×	×
Óskarsdóttir et al. [10]	√	Friendship	Degree centrality
Han et al. [36]	√	Guild, Trade (macro-level)	GCN embedding
Ours	√	Friendship, PvE teammate (micro-level)	MLNRL embedding

In summary, while existing research has made significant strides in game churn prediction by utilizing demographic and behavioral data, the integration of complex social structures remains in its infancy. Current relational approaches often rely on oversimplified graph metrics or focus exclusively on macro-level socio-economic ties, thereby neglecting the nuanced, micro-level interactions between players. To bridge this gap, this study proposes a novel churn prediction framework that captures the multiplex social influence inherent in gaming communities. By jointly modeling explicit friendship networks and implicit PvE teammate relationships through advanced network embeddings, our approach extracts the higher-order structural insights that traditional methods overlook, establishing a comprehensive paradigm for player churn prediction.

### 2.3 Influence of multiplex relationships and multiplex social influence

Numerous studies have demonstrated that relationship information is closely associated with individual behavioral outcomes. From a psychological perspective, prior studies indicate that interpersonal relationships influence individual behavior through three key mechanisms: social contagion, emotional transmission, and developmental processes. In real-world settings, individuals are typically embedded in multiple types of relationships—structures formally defined as multiplex relationships [41]. For instance, students may simultaneously share ties as classmates and as friends. Likewise, in online social networks, there exist multiplex relationships between netizens. On professional platforms such as LinkedIn, users can establish connections through business relationships or knowledge sharing relationships to facilitate information exchange [17]. Importantly, distinct types of relationships can exert differential effects on individual behavior. Empirical evidence suggests that multiplex relationships often have divergent effects, influencing behavioral outcomes in both positive and negative ways. Brass et al. [42] found that employees within organizations commonly maintain multiplex relationships—such as colleague, friendship, and neighborhood relationships—and that such multiplex relationships can constrain unethical behavior. Conversely, Shah et al. [43] reported that multiplex relationships with too many types of ties may impair employee performance. A plausible explanation is that multiplex relationships can blur social and professional boundaries, thereby inducing role ambiguity and psychological anxiety [44]. Collectively, these findings underscore the importance of accounting for the influence of multiplex relationships when modeling individual behavior in social networks, as multiplex relationships can generate complex and sometimes competing social influences.

How, then, do multiplex relationships influence individual behavior? One plausible mechanism is multiplex social influence. Social influence refers to the process through which an individual’s behavior is affected by attitudes, decisions, or behaviors of others within a social network [45]—a phenomenon that has been extensively documented in the literature. For example, an individual’s health behavior can be significantly influenced by others in their social network [46]. In recent years, increasing attention has been paid to multiplex social influence, which captures the extent to which individuals are simultaneously connected through multiple types of social interactions. Multiplex social influence can exert compound effects. For instance, Zagenczyk et al. [47] demonstrated that multiplex social influence alters employees’ leader–member exchange perceptions within networks combining colleague and friendship ties. Allen et al. [48] found that multiplex social influence promotes cooperative behavior in social dilemmas embedded in multiplex social networks consisting of both economic and social interactions. Recently, Guo et al. [8] showed that multiplex social influence jointly affects players’ time investment and monetary spending in online games.

Together, these findings highlight multiplex social influence as a critical mechanism through which such multiplex relationships affect individual behavior, thereby underscoring its importance for player churn prediction within online game networks.

### 2.4 Network embedding

Traditional approaches to modeling social influence typically rely on classical graph-theoretic metrics, which capture specific structural properties of nodes, such as centrality measures and clustering coefficients [49]. However, such metrics suffer from several notable limitations. First, classical graph-theoretic metrics capture only isolated aspects of social network structure, whereas social influence is inherently shaped by multi-scale information within the social network. Both direct influence from first-order interactions and indirect influence propagated through the network coexist within the social network [50,51]. Cao et al. [52] further showed that individuals with *k*-order proximity can exert influence on one another; and the influence can emerge among individuals belonging to the same community [53]. Second, the computational complexity of certain graph-theoretic metrics poses significant challenges. For instance, betweenness centrality—one of the most widely used centrality measures—exhibits cubic time complexity with respect to the number of nodes, rendering it impractical for large-scale networks [11]. These findings suggest that the traditional metrics are insufficient to fully characterize social influence.

Network embedding (also known as network representation learning) aims to learn node embeddings in low-dimensional latent space, preserving multi-scale, nonlinear structural properties or high-order structural proximities of networks. Moreover, a variety of scalable network embedding algorithms have been developed, enabling efficient learning on large-scale networks. The resulting embeddings are readily applicable to downstream machine learning tasks [12,13]. Owing to these advantages, network embedding has been increasingly adopted as an effective approach for modeling social influence. Qiu et al. [54] employed graph convolutional networks to learn node embeddings that capture influence-related structural dependencies, and subsequently used these embeddings to predict social influence outcomes including churn behavior. Wu et al. [55] proposed a framework that combines random walk–based neighborhood sampling with autoencoders to learn network embeddings capable of preserving multi-scale social influence, including both local and global structural patterns. Similarly, Zhao et al. [56] incorporated traditional network statistics (e.g., centrality measures) and ego-network structures as inputs to graph convolutional networks (GCNs), enabling the learned embeddings to represent multi-scale social influence beyond first-order connections. Furthermore, spatial aggregation models like GraphSAGE [57] have been introduced to inductively generate embeddings by sampling and aggregating features from a node’s local neighborhood.

However, despite their popularity, these mainstream embedding methods often present specific limitations when applied to highly dense and complex game communities. Random walk-based methods (e.g., DeepWalk [58]) primarily emphasize local proximities and often fail to capture the hierarchical macro-structures of a network. Conversely, standard graph neural networks (e.g., GCN and GraphSAGE) are notoriously prone to the over-smoothing problem in dense social networks, where repeated aggregations cause the embeddings of diverse nodes to become indistinguishable, thereby diluting nuanced behavioral signals.

To overcome these algorithmic constraints, we specifically adopt a network embedding algorithm called multi-scale structural information-based Laplacian generative adversarial network representation learning (MLNRL) [59] in this study. A critical advantage of our adopted framework lies in its exceptional computational scalability for massive gaming networks. Explicitly computing the foundational Shifted Positive Pointwise Mutual Information (SPPMI) matrix typically incurs a prohibitive cubic time complexity $O({\left|V\right|}^{\text{3}})$, rendering it unscalable for large-scale player graphs. To circumvent this problem, the structural prior information in our framework is implicitly approximated through efficient random walk simulations combined with a negative sampling skip-gram architecture. This mathematical equivalence strictly limits the computational cost to a linear scale [60]. Unlike standard GCN or random-walk methods, MLNRL is uniquely designed with a Laplacian generative adversarial cascade mechanism. This architecture effectively prevents over-smoothing while explicitly preserving multi-scale structural information—ranging from micro-level direct ties to macro-level community structures. Consequently, MLNRL is exceptionally well-suited to extract the complex, hierarchical dynamics inherent in gaming networks.

Overall, network embedding provides an effective proxy for social influence due to its ability to preserve complex structural patterns and its strong scalability. However, existing studies primarily focus on modeling social influence within a single type of network, neglecting its original multiplicity, and have not explicitly leveraged advanced network embedding to represent multiplex social influence in a multiplex social network. This gap suggests a critical opportunity to extend sophisticated network embedding techniques, such as MLNRL, into multiplex social networks, thereby holistically capturing multiplex social influence and substantially improving player churn prediction performance.

## 3 Materials and methods

**Fig 1:**
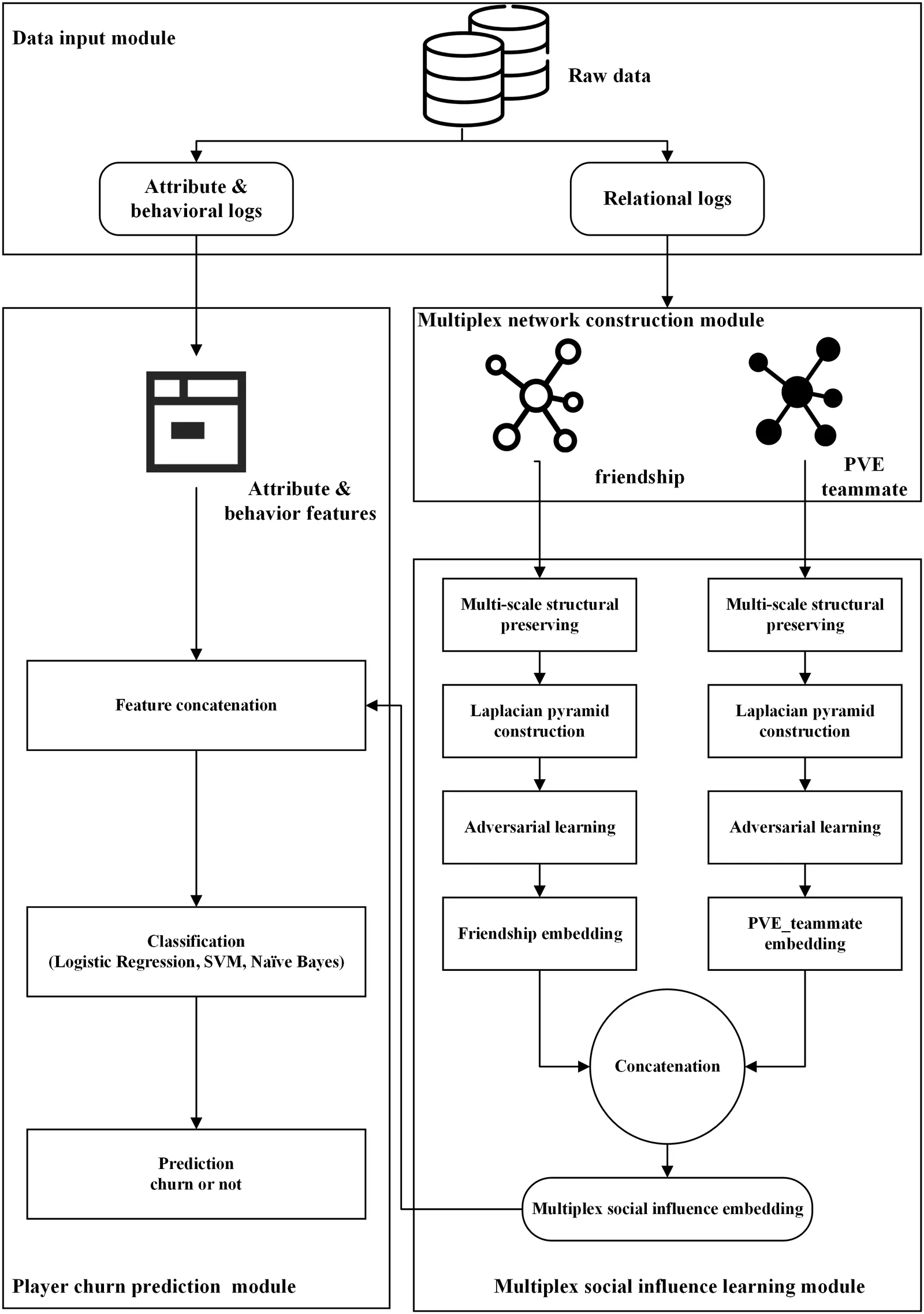
Multiplex social influence based player Churn prediction framework.

In this paper, we propose a novel framework—Multiplex Social Influence framework—to enhance the prediction performance of player churn prediction in online games. As illustrated in Fig 1, the proposed framework consists of four modules: Data Input Module, Multiplex Social Network Construction Module, Multiplex Social Influence Learning by MLNRL Module, and Player Churn Prediction Module. Each of these steps is described in detail below.

### 3.1 Data input module

As illustrated at the top of Fig 1, the framework begins with the Data Input Module. This module ingests raw game data and bifurcates it into two essential pipelines: (1) Attribute and Behavioral Logs, and (2) Relational Logs. The former captures individual-level profiling and engagement metrics, which are later processed into flat feature vectors for standard classification. The latter extracts the interactive traces between players, serving as the raw structural foundation for building explicit (friendship) and implicit (PvE teammate) social networks. This explicit separation ensures that both individual behaviors and multi-dimensional social influences are accurately captured and prepared for downstream modeling.

### 3.2 Multiplex social network construction module

In this step, we first collect the attribute, behavioral, and relationship data of online game players, and then construct a multiplex social network corresponding to different types of relationships among players. Specifically, we denote the churn behavior (e.g., whether a player churns) by Y, and represent players’ demographic attributes and in-game behavioral features by X. In addition, we model the multiplex social network using a set of undirected networks $G=\{G^{\left(\text{1}\right)},..,G^{\left(r\right)},\ldots ,G^{\left(R\right)}\}$, where each network $G^{\left(r\right)}=(\text{V},E^{\left(r\right)})$ corresponds to a relationship network constructed from a specific type of relationship r, and $V=\{v_{\text{1}},\ldots ,v_{n}\}$ denotes the set of players, and $E^{\left(r\right)}$ represents the set of edges induced by relationship r. Under these settings, the player set remains identical across all networks, while the number of networks is determined by the number of distinct relationship types, thereby collectively forming a multiplex social network.

### 3.3 Multiplex social influence learning module

**Fig 2:**
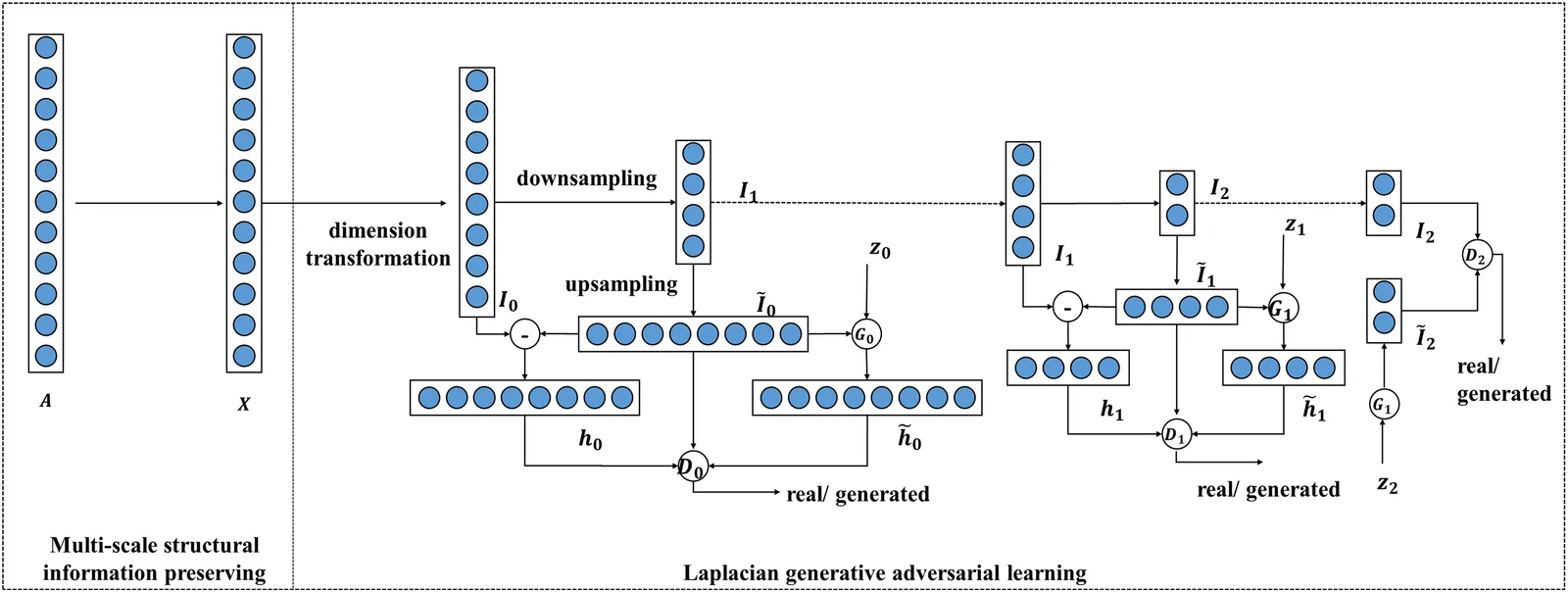
Framework of MLNRL [59].

In this step, we utilize the multiplex social network to learn network embeddings that capture multiplex social influence. Specifically, we adopt MLNRL method [59] to learn node embeddings for each relationship network. As shown in Fig 2, MLNRL comprises two core components: (1) the multi-scale structural information preserving component, which computes a shift positive pointwise mutual information (SPPMI) matrix to encode multi-scale structural information; and (2) the Laplacian generative adversarial learning component, which employs a Laplacian pyramid and generative adversarial networks to generate robust and informative node representations. By integrating these two components, MLNRL produces network embeddings that effectively preserve rich multi-scale structural information for each social network, thereby establishing a solid foundation for modeling social influence.

In this manner, we obtain the social influence received by node $v_{i}$ in the r-th relation network $G^{\left(r\right)}$, which is represented by its learned network embedding ${\hat{z}}_{i}^{\left(r\right)}$. Finally, as formalized in Equation (1), we aggregate the different social influences derived from different relationship types to construct the multiplex social influence of player $v_{i}$, which is achieved by concatenating the embeddings of $v_{i}$ learned from R relationship networks.


$$\begin{array}{c} {\hat{z}}_{i}=Concat\left({\hat{z}}_{i}^{\left(1\right)},\ldots ,{\hat{z}}_{i}^{\left(r\right)},\ldots ,{\hat{z}}_{i}^{\left(R\right)}\right) \end{array}$$ (1)

### 3.4 Player churn prediction module

In this step, we integrate the players’ demographic attributes and in-game behavioral features with the concatenated embeddings that capture multiplex social influence, and then input the combined feature set into player churn prediction models. Since the primary objective of this study is to examine whether incorporating multiplex social influence can enhance player churn prediction performance, the specific choice of paradigm—such as supervised machine learning models or survival analysis techniques—is not critical. Accordingly, we formulate churn prediction as a binary classification task and adopt three widely used classifiers for implementation: LR, SVM, and Gaussian naïve Bayes (GNB).

Formally, clf(·) denotes a certain binary classifier. The baseline player churn prediction model that relies solely on players’ demographic attributes and behavioral features can be expressed as Equation (2), where X denotes players’ demographic attributes and behavioral features. In contrast, the proposed framework, which incorporates both demographic attributes and behavioral features as well as multiplex social influence, is formulated in Equation (3), where$\;\hat{Z}$ is the matrix of multiplex social influence for all players, and $\hat{Z}={\hat{(z}}_{1},\ldots ,{\hat{z}}_{n})$.


$$\begin{array}{c} Y=clf\left(X\right) \end{array}$$ (2)

$$\begin{array}{c} Y=clf\left(X,\;\hat{Z}\right) \end{array}$$ (3)

Thus, if the prediction performance of Equation (3) outperforms that of Equation (2), this provides empirical evidence that incorporating multiplex social influence improves player churn prediction performance. Moreover, such results would indicate that multiplex social influence indeed exerts an effect on players’ churn behavior in online games.

### 3.5 Computational complexity analysis

To demonstrate the applicability of our proposed framework on massive real-world online game datasets, we provide a theoretical analysis of its computational complexity. Let n=|V| denote the number of players (nodes) and d denote the final embedding dimension, where d≪n.

Initially, computing the exact dense SPPMI matrix requires matrix multiplications, which incurs an intractable time complexity of $O(n^{\text{3}})$. Such a cubic complexity is strictly prohibitive for a real-world online game network. However, as detailed in the Literature review, our framework bypasses this by implicitly factorizing the SPPMI matrix via random walk simulations and the SGNS architecture, successfully reducing the complexity of the structural prior extraction to O(n).

Subsequently, for MLNRL algorithm, the Laplacian pyramid construction involves downsampling and upsampling operations whose time complexity is O(j) when processing a j-dimensional vector. Thus, constructing a (K+1)-layer Laplacian pyramid yielding a final d-dimensional embedding takes $O(\text{2}d({\text{2}}^{K}-\text{1}))$ time. Given that K is typically a small constant (e.g., between 1 and 10), the pyramid construction complexity scales as O(d).

Finally, during the adversarial learning phase, the backpropagation complexity for the transformation layer $f_{W}$ is proportional to its parameter size [61], i.e., $O\left(|W|\right)=O({\text{2}}^{K}dn)$. Because d≪n and K is a small constant, this strictly approximates to O(n). Similarly, the backpropagation complexity for the Laplacian GAN cascades is $O(n_{epoch}\times (n_{D}\times |\theta |+|\varphi |+|W|))$, where $n_{epoch}$ is the number of epochs, $n_{D}$ is the discriminator update frequency per epoch, and |θ| and |φ| are the parameter sizes of the generators and discriminators. Because the Laplacian pyramid causes the hidden layers to be exponentially smaller than the input layers, the total backpropagation complexity is remarkably reduced to O(n).

In summary, by replacing standard autoencoders with a Laplacian pyramid (which drastically reduces trainable parameters) and employing implicit SPPMI factorization, the overall time complexity of our embedding framework is O(n). This linear complexity strictly guarantees that our framework is highly scalable and computationally efficient for processing massive-scale game networks involving tens of millions of players.

## 4 Experiments and results

In this section, we conduct comparative experiments to assess the prediction performance of the proposed framework using a real-world online game dataset. This evaluation aims to demonstrate both the effectiveness of the proposed framework and the rigor of our study.

### 4.1 Data

This study leverages a dataset provided by a Chinese online game company that operates under a freemium business model, in which players can access the game free of charge and optionally purchase in-game items or advanced equipment to enhance their characters’ capabilities. A key feature of the game is its integrated social network-like friend system. Within this system, players may apply to become friends by sending friend requests to other players; once a request is accepted, the connected players can communicate with each other and observe one another’s in-game activities, including gameplay, purchasing, and payment behaviors. Additionally, the game offers a PvE mode, in which players team up to challenge in-game monsters and earn rewards such as experience points, items, and equipment. The PvE teammate relationship formed through PvE activities can also be represented as a dynamic social network.

The dataset comprises daily player-level attribute and behavioral records for 13,377,792 players collected between January 1 and March 31, 2011. These records include players’ login, gameplay, and payment behaviors. Furthermore, the dataset contains time-varying relationship information, capturing players’ daily friendship status (i.e., whether two players are friends in the game on a given day) as well as their PvE teammate relationships (i.e., whether two players participated in PvE activities together on a given day).

Although the raw game database contains logs for over 13 million registered accounts, a significant portion consists of isolated players or inactive accounts lacking any social ties. To ensure the quality of the structural representation and eliminate noise, we applied standard complex network filtering techniques by extracting the Largest Connected Component (LCC) from the raw network. The resulting dense multiplex social network comprises approximately 50,000 highly interactive players, providing a robust and representative foundation for evaluating social influence.

### 4.2 Data pre-processing

#### 4.2.1 Definition of active high-value players.

Existing literature consistently highlights the critical importance of high-value players to game companies. In the video game industry, such players are commonly referred to as “whales,” as they tend to contribute disproportionately large amounts of spending in the game [6]. For instance, Runge et al. [5] reported that the top 15% of players accounted for more than 60% of total revenue in casual social games, and Bertens et al. [34] found that high-value players contributed over half of the revenue in mobile social games. Given their significance for game company profits, this study specifically focuses on predicting churn behavior among high-value players.

**Fig 3:**
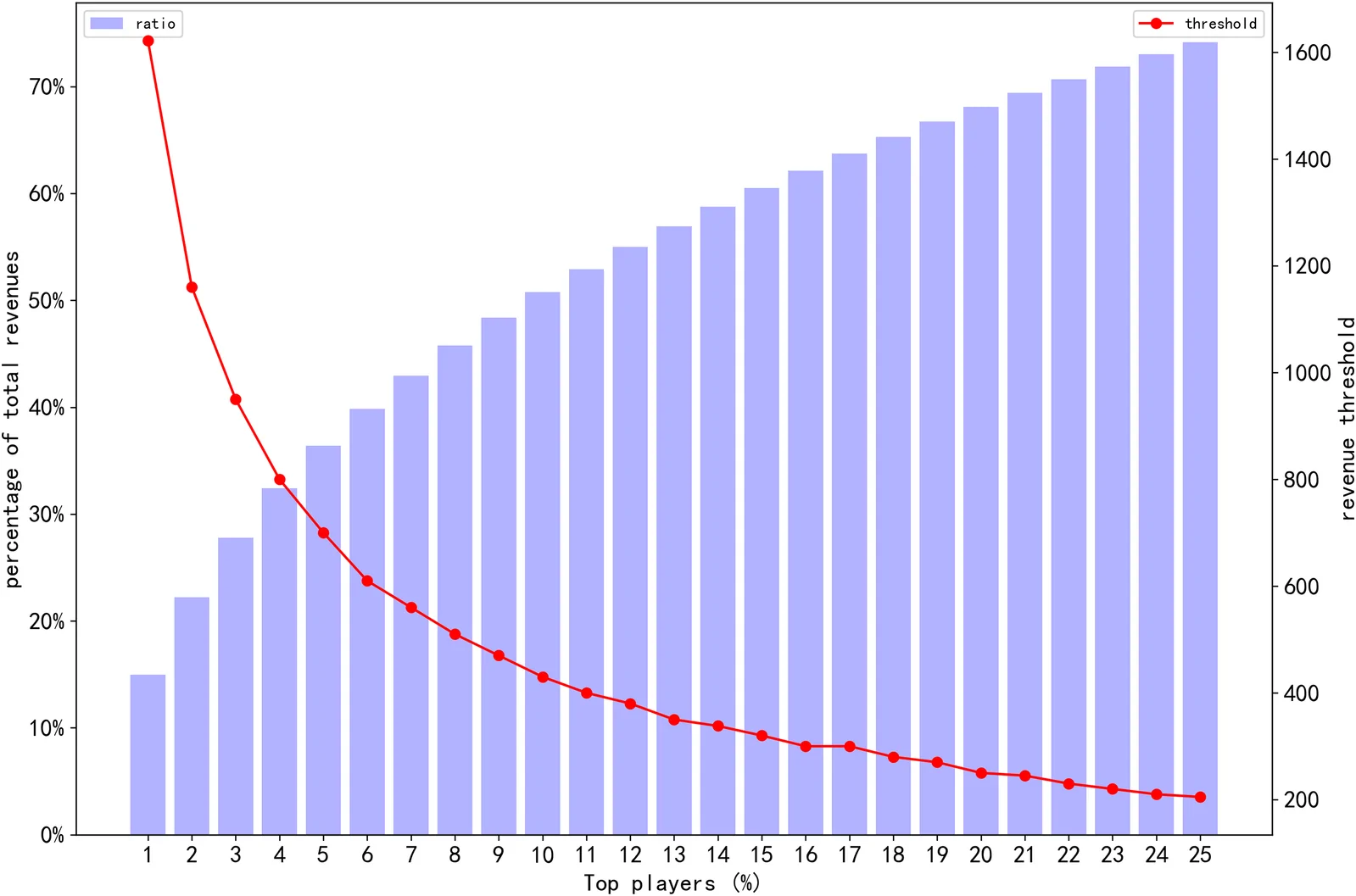
Contribution of top percentile paying players to total revenues.

To precisely define “high-value players,” we analyze the contributions of paying players to the total revenue within the game, following the approaches outlined by Perianez et al. [6]. The results, presented in Fig 3, illustrate the correlation between players’ spending and the overall revenue distribution. The horizontal axis represents the top percentiles of players based on their game spending; the left vertical axis shows the proportion of total game revenue generated by these players; the right vertical axis indicates the minimum spending threshold required for a player to be classified within each top percentile. The analysis reveals that the top 20% of players contribute more than 70% of the total revenue. Moreover, the minimum spending threshold for players in the top percentiles gradually flattens out, particularly for the top 20% and beyond. Based on these findings, we define high-value players at time t=0 as those in the top 20% of all game spending in the first 30 days before t=0.

Furthermore, to enable timely interventions, game companies aim to predict churn before it occurs and churn prediction should target active high-value players. In this study, we define active high-value players at time t=0 as those who are high-value players and have logged into the game at least once in the previous 7 days. Active high-value players represent players who not only contribute significantly to the revenue but also maintain recent engagement with the game.

#### 4.2.2 Definition of player churn.

Traditional player churn prediction studies have largely focused on subscription-based games, where churn can be clearly identified through the formal termination of a subscription contract. However, the freemium model has recently become the dominant revenue model for games such as mobile social games and MMORPGs, under which the relationship between players and game providers is non-contractual. Consequently, player churn cannot be identified through an explicit act of contract termination, but must instead be inferred from players’ disengagement behaviors. Meanwhile, prolonged player inactivity can have substantial negative consequences for game companies, including revenue loss and weakened network effects. As a result, in practical settings, player churn is commonly operationalized by defining a threshold of consecutive inactive days, beyond which a player is considered to have churned [28]. In this study, an inactive day is explicitly defined as a day on which a player does not log into the game. For example, if an active high-value player logs in on days t=1, t=3, and t=7, the player experiences two inactivity periods: one lasting 1 day (Δt=3−1−1=1) and another lasting 3 days (Δt=7−3−1=3.

**Fig 4:**
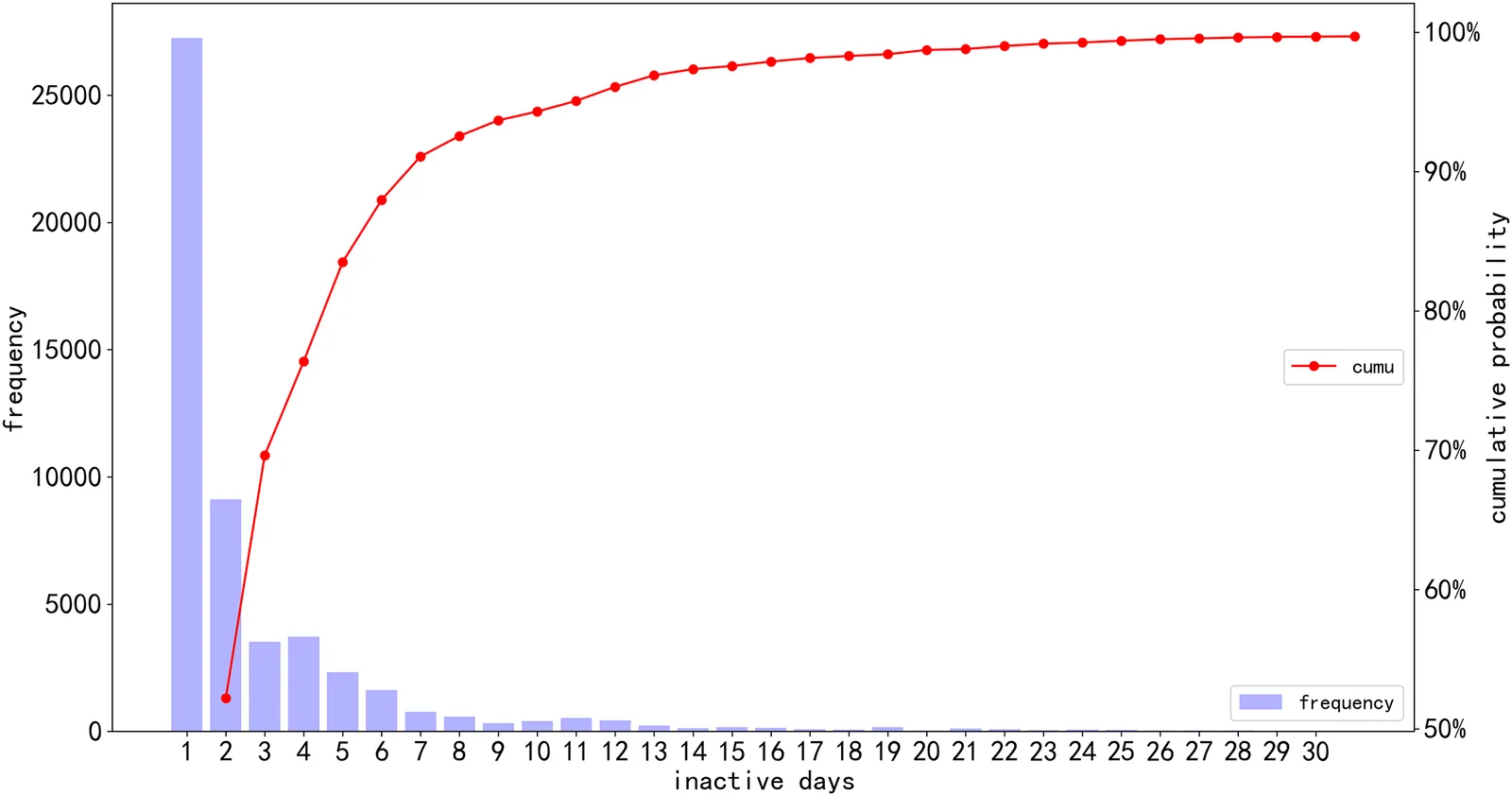
Inactive days distribution chart.

To determine an appropriate churn threshold, we compute all inactivity periods for active high-value players and visualize their distribution in Fig 4. For clarity, only inactivity periods of 30 days or fewer are shown, as longer inactivity durations occur infrequently in the dataset. The horizontal axis represents the number of inactive days, the left vertical axis denotes the frequency of inactivity periods, and the right vertical axis shows the cumulative distribution probability. The results indicate that over 90% of inactivity occurrences last between 1 and 7 days, while the probability of inactivity periods beyond 7 days is less than 10%. Based on this empirical distribution of inactivity periods, we assume that an active high-value player has churned if the active high-value player remains inactive for at least seven consecutive days. Specifically, we define the churn behavior of an active high-value player at time t as follows: the player fails to log in to the game for seven consecutive days, starting from any day between t and t+7. This definition aligns with industry standards and provides a practical, data-driven criterion for identifying churn behavior in free-to-play online games

#### 4.2.3 Construction of multiplex social networks.

Based on the daily friendship information and the teammate relationship information in PvE activities of players, we construct daily friendship networks and daily PvE teammate networks, respectively.

**Fig 5:**
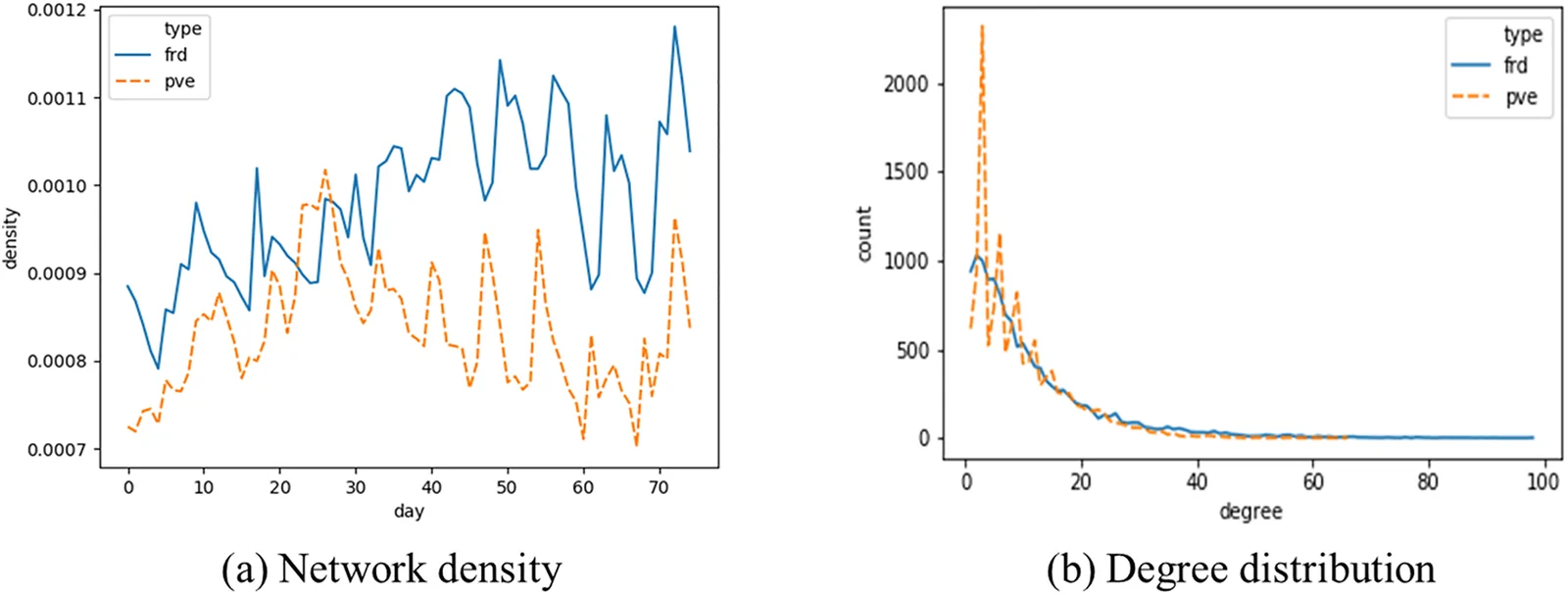
Statistics of two networks: (a): network density; (b) degree distribution.

To demonstrate that the two relationship networks are distinct, we compare their densities within 90 days (Fig 5(a)) as well as their node degree distributions (Fig 5(b)). The results show that the friendship network exhibits a higher density than the PvE teammate network, indicating that, on average, the number of friends per player is greater than that of PvE teammates. The degree distributions further reveal a difference between the two networks. Node degrees in the PvE teammate network are predominantly low, with most players having 10 or fewer teammates, whereas the friendship network exhibits a more gradual and stable decline in node degree frequency distribution. This indicates that node degrees in the friendship network are more tightly concentrated than those in the PvE teammate network. These results partially confirm the differences between the friendship network and the PvE teammate network. And multiplex social influence generated from multiplex social networks may aggregate different sources of social influence and improve the performance of player churn prediction.

#### 4.2.4 Multiplex social influence generation.

Since network embeddings are capable of capturing the social influence received by players, we employ them as proxy variables for players’ relationship information. As described in Section 2.4, the MLNRL algorithm is able to learn multi-scale structural information of nodes and demonstrates strong robustness. Accordingly, we apply the MLNRL algorithm to learn network embeddings for each player based on each type of network. The resulting embeddings are then concatenated to form a unified representation, which serves as the final embedding and captures the multiplex social influence for each player.

### 4.3 Variables

After the data preprocessing described in Section 4.2, we obtain the final dataset used for prediction, which includes churn labels, player demographic attributes, in-game behavioral features, and network embeddings. In addition, for the behavioral features, we compute the average values over the preceding 7 days for each variable to mitigate the impact of outliers. This allows the dataset to more accurately reflect players’ typical behavioral patterns. Brief descriptions and descriptive statistics are shown in Table 2 and Table 3, respectively.

**Table 2:** Data descriptions.

Data dimension	Variable	Description
Churn behavior	Churn	Whether a player will churn or not
Attribute	Gender	Player’s daily character gender
	Tenure	The number of days a player has registered
	Level	Player’s daily character level
Behavior	Coinspending	Player’s daily in-game coin spending amount
	Coinreward	Player’s daily in-game coin reward amount
	Coinexchange	Player’s daily in-game coin exchange amount
	Logduration	Player’s daily play time
	Logfrequency	Player’s daily login frequency
	Levelup	Player’s daily character upgrade level
	Payment	Player’s daily paying amount
	Payfrequency	Player’s daily paying frequency
	Coinspending_avg	Average amount of player’s in-game coin spending amount in the previous 7 days
	Coinreward_avg	Average amount of player’s in-game coin reward amount in the previous 7 days
	Coinexchange_avg	Average amount of player’s in-game coin exchange amount in the previous 7 days
	Logduration_avg	Average play time of players in the previous 7 days
	Logfrequency_avg	Average login frequency of players in the previous 7 days
	Levelup_avg	Average upgrade level of players in the previous 7 days
	Payment_avg	Average paying amount by players in the first 7 days
	Payfrequency_avg	Average paying frequency by players in the first 7 days
Relationship	${\hat{z}}_{i}^{\left(frd\right)}$	Network embedding that reflects a player’s social influence in daily friendship network
	${\hat{z}}_{i}^{\left(pve\right)}$	Network embedding that reflects a player’s social influence in daily friendship network
	${\hat{z}}_{i}$	Embedding that reflects a player’s multiplex social influence

**Table 3:** Descriptive statistics.

Variable	Mean	Standard deviation	Minimum	Maximum
Churn	0.032	0.18	0	1
Gender	0.34	0.47	0.00	1.00
Tenure	95.96	49.94	36.00	238.00
Level	39.50	2.10	19.00	40.00
Coinspending	$-\text{3}.\text{36}\times {\text{1}\text{0}}^{\text{6}}$	$\text{1}.\text{40}\times {\text{1}\text{0}}^{\text{5}}$	$-\text{1}.\text{41}\times {\text{1}\text{0}}^{\text{8}}$	0.00
Coinreward	$\text{7}.\text{80}\times {\text{1}\text{0}}^{\text{4}}$	$\text{1}.\text{40}\times {\text{1}\text{0}}^{\text{5}}$	0.00	$\text{1}.\text{55}\times {\text{1}\text{0}}^{\text{6}}$
Coinexchange	$\text{2}.\text{86}\times {\text{1}\text{0}}^{\text{6}}$	$\text{6}.\text{99}\times {\text{1}\text{0}}^{\text{6}}$	0.00	$\text{7}.\text{68}\times {\text{1}\text{0}}^{\text{7}}$
Logduration	237.58	202.05	0.00	1917.78
Logfrequency	3.06	2.21	0.00	24.00
Levelup	0.05	0.22	0.00	2.00
Payment	12.20	63.86	0.00	1120.00
Payfrequency	0.211	1.027	0.00	16.00
Coinspending_avg	$-\text{3}.\text{45}\times {\text{1}\text{0}}^{\text{6}}$	$\text{5}.\text{41}\times {\text{1}\text{0}}^{\text{6}}$	$-\text{5}.\text{05}\times {\text{1}\text{0}}^{\text{7}}$	0.00
Coinreward_avg	$\text{8}.\text{49}\times {\text{1}\text{0}}^{\text{4}}$	$\text{7}.\text{50}\times {\text{1}\text{0}}^{\text{4}}$	0.00	$\text{5}.\text{14}\times {\text{1}\text{0}}^{\text{5}}$
Coinexchange_avg	$\text{2}.\text{78}\times {\text{1}\text{0}}^{\text{6}}$	$\text{4}.\text{21}\times {\text{1}\text{0}}^{\text{6}}$	0.00	$\text{4}.\text{47}\times {\text{1}\text{0}}^{\text{7}}$
Logduration_avg	243.29	149.93	1.29	1048.02
Logfrequency_avg	3.11	1.48	1.00	10.29
Levelup_avg	0.18	0.40	0.00	2.00
Payment_avg	82.31	117.21	0.00	802.50
Payfrequency_avg	1.36	2.01	0.00	16.00

### 4.4 Prediction models and evaluation metrics

Based on the research framework described in Section 3 and the pre-processed dataset, we employ different churn prediction models listed in Table 4 to predict player churn behavior and conduct comparative evaluations to demonstrate that incorporating multiplex social influence improves prediction performance. Specifically, Equation (8), referred to as *multiplex_social_influence* model (MSIM), implements the proposed framework in this study by incorporating players’ attribute, behavioral features, and multiplex social influence. To demonstrate the superiority of the proposed framework, five additional models are introduced for comparative evaluation. Equation (4) represents the *baseline* model (BSM), which relies solely on players’ attribute and behavioral features for churn prediction. Equation (5) (*friendship* model, FRDM) and Equation (6) (*PvE_teammate* model, PVEM) extend BS by incorporating network embeddings that capture social influence in the friendship network (${\hat{Z}}^{\left(frd\right)}$) and the PvE teammate network (${\hat{Z}}^{\left(pve\right)}$), respectively. Furthermore, to verify that performance improvements stem from social influence or multiplex social influence—rather than from increased feature dimension—Equation (7) (*white_noise_1* model, WN1M) and Equation (9) (*white_noise_2* model, WN2M) replace the corresponding network embeddings with white-noise vectors of identical dimensions.

**Table 4:** Different churn prediction models.

Model	Model Abbreviation	Equation	No
Baseline	BSM	Y=clf(𝐗)	(4)
Friendship	FRDM	$Y=clf\left(𝐗,\;{\hat{Z}}^{\left(frd\right)}\right)$	(5)
PvE_teammate	PVEM	$Y=clf\left(𝐗,\;{\hat{Z}}^{\left(pve\right)}\right)$	(6)
white_noise_1	WN1M	Y=clf(𝐗, e1)	(7)
multiplex_social_influence	MSIM	$Y=clf\left(𝐗,\;\hat{Z}\right)$	(8)
white_noise_2	WN2M	Y=clf(𝐗, e2)	(9)

Based on the comparative evaluation of the six models described above, if the prediction performance of either FRDM or PVEM exceeds that of both BSM and WN1M, this would indicate that social influence within a specific social network contributes meaningful predictive power to player churn prediction, and that the corresponding network embeddings effectively capture the structural information pertinent to such influence. More significantly, if MSIM outperforms all five other models, this would serve as strong evidence that multiplex social influence yields superior prediction performance compared with any single type of social influence.

To evaluate the performance of the churn prediction models, we adopt five widely recognized evaluation metrics based on the confusion matrix (Table 5): accuracy, precision, recall, F1-score, and the Area Under the Receiver Operating Characteristic Curve (AUC). Accuracy, defined in Equation (10), measures the proportion of correctly classified samples among all test instances. While it serves as a fundamental benchmark for overall classification capabilities, a higher accuracy generally signifies superior global classification performance. Precision, as formulated in Equation (11), reflects the proportion of truly churned players among all instances predicted as churned by the model. This metric underscores the exactness of the classifier, ensuring that retention resources are not squandered on loyal players. Conversely, recall, expressed in Equation (12), quantifies the fraction of actual churned players successfully identified by the model. By measuring the classifier’s completeness, a higher recall mitigates the risk of missing critical opportunities to retain valuable players. In practice, precision and recall often exhibit an inherent trade-off, where optimizing one may lead to the degradation of the other. To address this, the F1-score is employed as the harmonic mean of precision and recall, yielding a value between 0 and 1. By jointly considering both dimensions, the F1-score provides a more balanced and comprehensive assessment, particularly in the presence of severe class imbalance. Finally, AUC evaluates the model’s capacity to distinguish between churned and non-churned players across various classification thresholds, where a larger AUC value indicates stronger and more robust discriminative capabilities.

**Table 5:** Confusion Matrix.

	Predicted Positive	Predicted Negative
Actual Positive	True Positive (TP)	False Negative (FN)
Actual Negative	False Positive (FP)	True Negative (TN)


$$\begin{array}{c} Accuracy=\frac{TP+TN}{TP+FN+FP+TN} \end{array}$$ (10)

$$\begin{array}{c} Precision=\frac{TP}{TP+FP} \end{array}$$ (11)

$$\begin{array}{c} Recall=\frac{TP}{TP+FN} \end{array}$$ (12)

$$\begin{array}{c} F\text{1}-score=\frac{\text{2}\cdot Precision\cdot Recall}{Precision+Recall}=\frac{\text{2}TP}{\text{2}TP+FP+FN} \end{array}$$ (13)

### 4.5 Experiment settings

First, we implement the MLNRL algorithm in Python to learn player-level network embeddings from the friendship network and the PvE teammate network, respectively. The embedding dimension for each network is set to 16; consequently, the embedding representing multiplex social influence has a dimensionality of 32.

Second, we select several widely used binary classification models, including LR, SVM with a linear kernel and a radial basis function (RBF) kernel, as well as GNB model. All classifiers are implemented using the Python third-party library *Sklearn* [62].

Finally, the pre-processed dataset contains 38 records corresponding to churned players and 1,142 non-churned players, resulting in a highly imbalanced distribution. This extreme skewness accurately reflects the real-world operational reality of online games, where only a tiny fraction of an active community typically churns within a strictly defined short-term window [5,63]. While this natural imbalance is a standard challenge in gaming analytics, as noted by Sun et al. [64], directly training classifiers on imbalanced data may lead to biased prediction results. To mitigate this issue, we apply the Synthetic Minority Over-sampling Technique (SMOTE), a widely adopted oversampling method, to balance the dataset [65]. The SMOTE algorithm is implemented using the Python library *imbalanced-learn* [66]. The dataset is then randomly split into training and test sets with a ratio of 3:2, such that the training set comprises 60% of the entire dataset. To enhance the robustness of the experimental results, we conduct 10 repeated experiments for each combination of classifier with feature set, and report the average value of each evaluation metric as the final performance outcome.

### 4.6 Results

Tables 6 reports the player churn prediction results obtained using LR, SVM (linear kernel), SVM (RBF kernel) and GNB, respectively. As shown in the table, across all four binary classifiers, both the single-layer social influence models—FRDM and PVEM—consistently achieve higher performance than BSM and WN1M. Quantitatively, using the SVM (RBF kernel) as an example, integrating friendship and PvE teammate networks improves the AUC from 0.9508 in BSM to 0.9749 and 0.9674, respectively. Furthermore, the inclusion of relationship data noticeably boosts the recall metric (e.g., increasing from 0.9634 in BSM to 0.9733 in PVEM and 0.9788 in FRDM), indicating a stronger ability to correctly identify actual churners with fewer false negatives. These confirm that relationship information indeed enhances churn prediction through social influence. Moreover, it indirectly demonstrates that the proposed MLNRL algorithm is highly capable of learning network embeddings that preserve players’ multi-scale structural information.

**Table 6:** Prediction results of four classification algorithms for each model.

Classification Algorithm	Model	Accuracy	Precision	Recall	F1-score	AUC
LR	BSM	0.8098	0.7774	0.8686	0.8203	0.8631
	FRDM	0.8137	0.7891	0.8652	0.8252	0.8666
	PVEM	0.8310	0.7883	0.9062	0.8430	0.8813
	WN1M	0.7968	0.7743	0.8487	0.8095	0.8516
	MSIM	**0.8444**	**0.7949**	**0.9259**	**0.8553**	**0.8891**
	WN2M	0.7907	0.7645	0.8299	0.7955	0.8509
SVM (linear kernel)	BSM	0.8089	0.7498	0.9218	0.8266	0.8575
	FRDM	0.8219	0.7610	0.9413	0.8415	0.8635
	PVEM	0.8411	0.7756	0.9587	0.8574	0.8668
	WN1M	0.8101	0.7605	0.9110	0.8288	0.8567
	MSIM	**0.8523**	**0.7883**	**0.9631**	**0.8667**	**0.8903**
	WN2M	0.7991	0.7505	0.9006	0.8184	0.8516
SVM (RBF kernel)	BSM	0.9022	0.8580	0.9634	0.9076	0.9508
	FRDM	0.9159	0.8693	0.9788	0.9207	0.9749
	PVEM	0.9153	0.8721	0.9733	0.9198	0.9674
	WN1M	0.7934	0.6293	0.6906	0.6579	0.7101
	MSIM	**0.9307**	**0.8914**	**0.9789**	**0.9330**	**0.9826**
	WN2M	0.5903	0.5875	0.6015	0.5934	0.6281
GNB	BSM	0.6046	0.5606	0.9926	0.7165	0.8355
	FRDM	0.6071	0.5640	**0.9948**	0.7198	0.8620
	PVEM	0.6171	0.5687	0.9907	0.7255	0.8184
	WN1M	0.6033	0.5568	0.9939	0.7161	0.8330
	MSIM	**0.6218**	**0.5723**	0.9920	**0.7257**	**0.8701**
	WN2M	0.5978	0.5528	0.9914	0.7097	0.8246

In addition, the detailed metric comparison reveals that PVEM generally exhibits slightly superior or highly competitive performance compared with FRDM (e.g., achieving higher accuracy and F1-scores in LR and SVM (linear kernel)). This suggests that the social influence arising from PvE teammate relationships plays a more prominent role in players’ churn decisions. A plausible explanation is that explicit “friendship” ties do not necessarily guarantee deep or continuous communication after users add each other; in contrast, PvE teammate relationships inherently require deepened interaction, mutual dependence, and frequent communication during collaborative tasks. Therefore, the behavioral patterns embedded in PvE relationships are more expressive and effective for churn prediction.

Finally, across all classifiers, MSIM significantly and consistently outperforms BSM, FRDM, PVEM, WN1M, and WN2M. Notably, MSIM reaches its peak performance under SVM (RBF kernel)—accuracy: 0.9307, precision: 0.8914, recall: 0.9789, F1-score: 0.9330, AUC: 0.9826. The simultaneous improvements in both precision and recall imply that the MSI framework not only captures more potential churners but also maintains a high level of prediction performance.

Based on these quantitative findings, the explicit contributions of our study are threefold. First, we empirically prove that utilizing the MLNRL algorithm to capture structural information from game networks is highly effective for behavioral prediction. Second, we demonstrate that aggregating different types of social influence (friendship and PvE teammates) into multiplex social influence yields a synergistic effect, providing substantially greater predictive benefits than relying on any single type of social network alone. Third, the high precision and recall achieved by our MSI framework offer significant practical value for the gaming industry, enabling operators to accurately target high-risk players for retention campaigns without wasting resources on false positives.

### 4.7 Ablation study

A potential critical question regarding our proposed MSI-based Player Churn Prediction framework is whether its superior prediction performance genuinely stems from the synergistic integration of multiple social interactions (i.e., friendship and PvE teammate relations), or merely from an artifact of increased feature dimensionality after concatenation.

To rigorously isolate the effect of information diversity from feature dimensionality, we designed a targeted ablation study. Specifically, we constructed two dimensionality-matched ablation baselines—FRDM+WN and PVEM+WN—in which only one type of true social influence embedding (either friendship or PvE) was retained and padded with randomly generated White Noise (WN) vectors of the exact same length as the missing social embedding. Consequently, both FRDM+WN and PVEM+WN possess the identical feature dimensionality to the complete MSIM, but contain only a single source of meaningful social information.

The results of this ablation study are presented in Table 7. Several compelling observations can be drawn. First, keeping the dimensionality constant, the complete MSIM overwhelmingly outperforms both FRDM+WN and PVEM+WN across all classifiers and metrics. For example, MSIM achieves an accuracy of 0.8400 and an AUC of 0.8816 under LR, significantly surpassing FRDM+WN (accuracy: 0.7973, AUC: 0.8427) and PVEM+WN (accuracy: 0.8005, AUC: 0.8567). These results clearly indicate that the performance gain of MSI framework is driven by the rich, complementary information provided by the multiplex social networks, rather than merely having a longer feature vector. Second, when compared to the pure single social influence models (FRDM and PVEM), the addition of white noise (FRDM+WN and PVEM+WN) yields either marginal changes in linear models or a drastic drop in complex models. Most notably, in the SVM (RBF kernel) classifier, replacing one true social embedding with noise causes the AUC to plummet from over 0.94 to approximately 0.63. In stark contrast, replacing the noise with the second true social embedding (i.e., forming the complete MSIM) skyrockets the AUC to a peak of 0.9703 with an F1-score of 0.9083.

**Table 7:** Ablation study results: prediction performance of four classification algorithms across models.

Classification Algorithm	Model	Accuracy	Precision	Recall	F1-score	AUC
LR	BSM	0.7825	0.7569	0.8446	0.7982	0.8325
	FRDM	0.8133	0.7691	0.8924	0.8259	0.8504
	PVEM	0.8112	0.7626	0.9002	0.8253	0.8616
	FRDM+WN	0.7973	0.7562	0.8685	0.8083	0.8427
	PVEM+WN	0.8005	0.7775	0.8510	0.8125	0.8567
	MSIM	**0.8400**	**0.7894**	**0.9312**	**0.8543**	**0.8816**
SVM (linear kernel)	BSM	0.7833	0.7235	0.9191	0.8095	0.8388
	FRDM	0.8123	0.7498	0.9262	0.8285	0.8630
	PVEM	0.8252	0.7577	0.9572	0.8457	0.8751
	FRDM+WN	0.8119	0.7554	0.9252	0.8316	0.8552
	PVEM+WN	0.8195	0.7585	0.9397	0.8392	0.8671
	MSIM	**0.8432**	**0.7793**	**0.9621**	**0.8610**	**0.8773**
SVM (RBF kernel)	BSM	0.8364	0.7704	0.9664	0.8571	0.9006
	FRDM	0.8916	0.8394	0.9698	0.8998	0.9576
	PVEM	0.8713	0.8050	0.9778	0.8829	0.9425
	FRDM + WN	0.5927	0.5909	0.6176	0.6023	0.6324
	PVEM+WN	0.5938	0.5959	0.5977	0.5947	0.6319
	MSIM	**0.9013**	**0.8445**	**0.9827**	**0.9083**	**0.9703**
GNB	BSM	0.5981	0.5571	0.9959	0.7144	0.7639
	FRDM	0.5991	0.5546	0.9930	0.7116	0.8408
	PVEM	0.6126	0.5635	0.9970	0.7200	0.7870
	FRDM+WN	0.6008	0.5546	0.9919	0.7113	0.8368
	PVEM+WN	0.6143	0.5618	0.9916	0.7172	0.8036
	MSIM	**0.6172**	**0.5707**	**0.9970**	**0.7258**	**0.8413**

In conclusion, the ablation study provides definitive empirical evidence that explicit friendship ties and implicit PvE teammate relations capture distinctly different but highly complementary aspects of user behavior. The necessity and superiority of modeling these dynamics as a multiplex social network are thus firmly validated.

### 4.8 Robustness check

#### 4.8.1 Different network embedding algorithms.

To further rigorously verify the generalizability of our MSI-based Player Churn Prediction framework, it is imperative to ensure that the observed performance gains are not strictly dependent on the specific use of the MLNRL algorithm. Therefore, we conducted a comprehensive robustness check by replacing MLNRL with three widely adopted, state-of-the-art network representation learning algorithms: DeepWalk (a random-walk based topological embedding), GCN (a spectral graph convolution approach), and GraphSAGE (a spatial domain inductive aggregation approach). We re-run the entire evaluation pipeline using the embeddings generated by these three alternative algorithms.

The prediction results utilizing DeepWalk, GCN, and GraphSAGE embeddings are presented in Tables 8, 9, and 10, respectively. Two critical conclusions can be drawn from these results. First, the fundamental superiority of our proposed framework remains highly consistent regardless of the underlying embedding algorithm. Across all three alternative methods (DeepWalk, GCN, and GraphSAGE), the complete MSIM consistently and significantly outperforms BSM, the noise-padded baselines (WN1M and WN2M), and the single social influence models (FRDM and PVEM) across almost all classifiers. For instance, using GraphSAGE embeddings under the SVM (RBF kernel) classifier, MSIM achieves an outstanding AUC of 0.9947, drastically higher than FRDM (0.9763) or PVEM (0.9754). These results strongly indicate that the predictive power of the MSI framework is an inherent structural property of the gaming community, rather than a coincidental byproduct of one specific embedding technique.

**Table 10:** Prediction results of four classification algorithms for each model using GraphSage embeddings.

Classification Algorithm	Model	Accuracy	Precision	Recall	F1-score	AUC
LR	BSM	0.7788	0.7460	0.8449	0.7920	0.8346
	FRDM	0.8052	0.7722	0.8658	0.8160	0.8690
	PVEM	0.7891	0.7613	0.8430	0.7998	0.8461
	WN1M	0.7630	0.7348	0.8274	0.7780	0.8197
	MSIM	**0.8198**	**0.7855**	**0.8837**	**0.8314**	**0.8816**
	WN2M	0.7553	0.7321	0.8052	0.7666	0.8140
SVM (linear kernel)	BSM	0.7769	0.7180	0.9161	0.8047	0.8294
	FRDM	0.8035	0.7492	0.9134	0.8229	0.8593
	PVEM	0.8023	0.7452	0.9174	0.8223	0.8516
	WN1M	0.7844	0.7368	0.9005	0.8102	0.8345
	MSIM	**0.8371**	**0.7831**	**0.9309**	**0.8505**	**0.8852**
	WN2M	0.7723	0.7224	0.8910	0.7976	0.8235
SVM (RBF kernel)	BSM	0.8355	0.7666	0.9643	0.8540	0.8978
	FRDM	0.9192	0.8773	0.9732	0.9227	0.9763
	PVEM	0.9203	0.8764	0.9794	0.9250	0.9754
	WN1M	0.6207	0.6149	0.6615	0.6363	0.6631
	MSIM	**0.9564**	**0.9272**	**0.9900**	**0.9575**	**0.9947**
	WN2M	0.5634	0.5638	0.5731	0.5662	0.5986
GNB	BSM	0.5967	0.5530	0.9947	0.7108	0.7696
	FRDM	0.6004	0.5558	0.9933	0.7126	0.8755
	PVEM	0.5967	0.5536	0.9954	0.7113	0.8822
	WN1M	0.5967	0.5517	0.9958	0.7100	0.7629
	MSIM	**0.5988**	**0.5552**	**0.9939**	**0.7124**	**0.9213**
	WN2M	0.5980	0.5525	0.9904	0.7092	0.7659

**Table 8:** Prediction results of four classification algorithms for each model using DeepWalk embeddings.

Classification Algorithm	Model	Accuracy	Precision	Recall	F1-score	AUC
LR	BSM	0.7534	0.7080	0.8523	0.7730	0.8003
	FRDM	0.8000	0.7563	0.8850	0.8150	0.8370
	PVEM	0.7707	0.7381	0.8433	0.7862	0.8216
	WN1M	0.7497	0.7152	0.8313	0.7682	0.7957
	MSIM	**0.8229**	**0.7672**	**0.9255**	**0.8386**	**0.8525**
	WN2M	0.7420	0.7207	0.7978	0.7568	0.7934
SVM (linear kernel)	BSM	0.7686	0.7066	0.9201	0.7990	0.8135
	FRDM	0.8174	0.7576	0.9463	0.8411	0.8438
	PVEM	0.7867	0.7264	0.9187	0.8110	0.8325
	WN1M	0.7590	0.7044	0.9010	0.7902	0.8074
	MSIM	**0.8307**	**0.7554**	**0.9751**	**0.8507**	**0.8623**
	WN2M	0.7626	0.7180	0.8759	0.7888	0.8056
SVM (RBF kernel)	BSM	0.8263	0.7564	0.9641	0.8475	0.8893
	FRDM	0.8969	0.8329	0.9893	0.9043	0.9612
	PVEM	0.8469	0.7897	0.9515	0.8629	0.9224
	WN1M	0.5899	0.5825	0.6316	0.6039	0.6299
	MSIM	**0.8976**	**0.8384**	**0.9850**	**0.9057**	**0.9683**
	WN2M	0.5536	0.5555	0.5604	0.5572	0.5665
GNB	BSMM	0.6040	0.5583	0.9986	0.7161	0.7598
	FRDM	0.6127	0.5633	0.9955	0.7192	0.8886
	PVEM	0.6199	0.5679	0.9968	0.7234	0.8766
	WN1M	0.6061	0.5551	0.9953	0.7126	0.7563
	MSIM	**0.6241**	**0.5723**	**0.9969**	**0.7269**	**0.9266**
	WN2M	0.6034	0.5559	0.9939	0.7129	0.7585

**Table 9:** Prediction results of four classification algorithms for each model using GCN embeddings.

Classification Algorithm	Model	Accuracy	Precision	Recall	F1-score	AUC
LR	BSM	0.7788	0.7460	0.8449	0.7920	0.8346
	FRDM	0.8041	0.7673	0.8798	0.8191	0.8520
	PVEM	0.8222	0.7862	0.8878	0.8336	0.8688
	WN1M	0.7630	0.7348	0.8273	0.7780	0.8197
	MSIM	**0.8411**	**0.8108**	**0.8945**	**0.8500**	**0.8900**
	WN2M	0.7553	0.7321	0.8052	0.7666	0.8140
SVM (linear kernel)	BSM	0.7769	0.7180	0.9160	0.8047	0.8293
	FRDM	0.8158	0.7613	0.9245	0.8347	0.8610
	PVEM	0.8336	0.7738	0.9444	0.8504	0.8725
	WN1M	0.7844	0.7368	0.9004	0.8101	0.8344
	MSIM	**0.8515**	**0.7829**	**0.9747**	**0.8682**	**0.8679**
	WN2M	0.7723	0.7224	0.8909	0.7975	0.8235
SVM (RBF kernel)	BSM	0.8355	0.7666	0.9643	0.8540	0.8978
	FRDM	0.8900	0.8348	0.9718	0.8980	0.9624
	PVEM	0.8866	0.8228	0.9842	0.8962	0.9461
	WN1M	0.6207	0.6149	0.6615	0.6363	0.6631
	MSIM	**0.9226**	**0.8731**	**0.9877**	**0.9267**	**0.9817**
	WN2M	0.5634	0.5638	0.5731	0.5662	0.5986
GNB	BSM	0.5967	0.5530	0.9947	0.7108	0.7696
	FRDM	0.6052	0.5620	0.9937	0.7179	0.8667
	PVEM	0.6037	0.5611	0.9915	0.7166	0.8419
	WN1M	0.5967	0.5517	0.9958	0.7100	0.7629
	MSIM	**0.6014**	**0.5560**	**0.9899**	**0.7120**	**0.8878**
	WN2M	0.5980	0.5525	0.9904	0.7092	0.7659

Second, although alternative graph algorithms demonstrate commendable performance, the MLNRL algorithm exhibits consistently superior and more stable predictive capabilities for this specific task. When cross-referenced with our primary findings (Table 6), MLNRL-generated embeddings achieve the highest or most competitive metrics across the majority of classifiers. Notably, under LR and SVM (linear kernel), the MLNRL-based MSIM reaches AUCs of 0.8891 and 0.8903, respectively—distinctly outperforming DeepWalk, GCN, and GraphSAGE.

This empirical superiority closely aligns with our theoretical rationale. Whereas DeepWalk is inherently constrained by local topological proximities, and standard spatial/spectral GNNs (e.g., GCN and GraphSAGE) are notoriously prone to the over-smoothing problem in dense social graphs, MLNRL leverages a unique Laplacian generative adversarial cascade. This mechanism explicitly preserves multi-scale structural information without signal degradation, making it exceptionally adept at capturing the complex, hierarchical dynamics inherent in gaming networks.

In summary, this robustness check serves a dual purpose: it empirically validates the universal applicability of the multiplex social influence framework across different embedding paradigms, while undeniably confirming that MLNRL remains the optimal algorithmic choice for extracting such social structures in churn prediction.

#### 4.8.2 Different embedding fusion methods.

In our proposed MSI-based Player Churn Prediction framework, we utilize the concatenation operation to fuse the embeddings derived from the friendship network and the PvE teammate network. To ensure that our conclusion regarding the superiority of the MSI-based Player Churn Prediction framework is robust and not merely an artifact of this specific fusion operation, we conduct a robustness check evaluating alternative embedding fusion strategies.

Specifically, we compare our primary concatenation approach, denoted as MSIM (C), with two widely used element-wise operations: MSIM (A), which applies element-wise addition to the two embeddings, and MSIM (M), which applies element-wise multiplication. The prediction performance of these different fusion strategies is detailed in Table 11.

**Table 11:** Prediction results of four classification algorithms for each model under different fusion strategies.

Classification Algorithm	Model	Accuracy	Precision	Recall	F1-score	AUC
LR	BSM	0.7826	0.7480	0.8551	0.7977	0.8275
	FRDM	0.8123	0.7793	0.8772	0.8252	0.8531
	PVEM	0.8190	0.7721	0.8991	0.8305	0.8701
	MSIM (C)	**0.8368**	**0.7865**	**0.9240**	**0.8494**	**0.8795**
	MSI M(A)	0.8200	0.7724	0.8995	0.8308	0.8676
	MSI M(M)	0.8284	0.7852	0.9036	0.8401	0.8709
SVM (linear kernel)	BSM	0.7766	0.7137	0.9222	0.8045	0.8344
	FRDM	0.8123	0.7588	0.9214	0.8320	0.8579
	PVEM	0.8276	0.7621	0.9559	0.8480	0.8733
	MSIM (C)	**0.8437**	**0.7721**	**0.9665**	**0.8584**	**0.8850**
	MSIM (A)	0.8209	0.7657	0.9246	0.8375	0.8736
	MSIM (M)	0.8172	0.7573	0.9365	0.8372	0.8752
SVM (RBF kernel)	BSM	0.8313	0.7610	0.9632	0.8501	0.8961
	FRDM	0.8830	0.8259	0.9698	0.8919	0.9554
	PVEM	0.8725	0.8133	0.9701	0.8846	0.9460
	MSIM (C)	**0.9074**	**0.8566**	**0.9800**	**0.9141**	**0.9718**
	MSIM (A)	0.8832	0.8270	0.9702	0.8927	0.9461
	MSIM (M)	0.8653	0.8084	0.9631	0.8789	0.9309
GNB	BSM	0.5933	0.5503	0.9934	0.7082	0.7608
	FRDM	0.6073	0.5625	0.9943	0.7185	0.8312
	PVEM	0.6121	0.5626	0.9972	0.7192	0.7863
	MSIM (C)	**0.6162**	**0.5679**	**0.9959**	**0.7232**	**0.8444**
	MSIM (A)	0.6107	0.5610	0.9969	0.7179	0.8217
	MSIM (M)	0.6438	0.5846	0.9863	0.7340	0.7978

Two significant findings emerge from this comparative analysis. First, the fundamental concept of multiplex social influence remains robust regardless of the fusion strategy. Across the majority of classifiers, all three fusion variants—MSIM (C), MSIM (A), and MSIM (M)—generally outperform BSM and the single social influence models (FRDM and PVEM). For example, under the LR classifier, both MSIM (A) and MSIM (M) yield higher F1-scores and AUCs than the isolated FRDM or PVEM. This finding reinforces our core hypothesis that integrating explicit and implicit social relationships provides a synergistic predictive advantage that holds true irrespective of the mathematical operator used for fusion.

Second, the concatenation operation emerges as the empirically optimal and most stable fusion strategy. As highlighted in Table 11, MSIM (C) consistently achieves the highest performance metrics across almost all scenarios. The performance gap is particularly evident in complex, non-linear classifiers. For instance, in the SVM (RBF kernel), MSIM (C) achieves a remarkable AUC of 0.9718, significantly outperforming MSIM (A) (0.9461) and MSIM (M) (0.9309). The theoretical justification for this outcome lies in the nature of the networks. Friendship and PvE teammate relations represent two heterogeneous dimensions of social behavior. Element-wise addition or multiplication enforces a hard, symmetric interaction between the components of the two vectors, which can lead to information loss, feature interference, or the over-smoothing of distinct behavioral signals. In contrast, concatenation preserves the complete, orthogonal feature spaces of both networks independently, allowing the downstream machine learning classifiers to flexibly learn the optimal weights for each social dimension.

In conclusion, this robustness check confirms that our proposed framework is highly resilient to algorithmic variations, while simultaneously validating that concatenation is the most effective mechanism for capturing the diverse behavioral insights embedded in multi-source social networks.

#### 4.8.3 Different churn definitions.

A potential concern regarding our primary dataset is the highly skewed class distribution (e.g., 38 churned vs. 1,142 non-churned players). First, it is crucial to note that extreme class imbalance is a well-documented and inherent characteristic of churn prediction in the video game industry, particularly in free-to-play or MMORPG environments. Existing literature emphasizes that active user bases are typically massive compared to the fraction of users who churn within a specific short-term observation window [5,63]. Therefore, tackling such imbalance is a standard challenge in real-world operational environments.

To rigorously validate the robustness and generalizability of our proposed approach against this imbalance, we conduct an additional experiment by varying the temporal thresholds used to define a “churner”. Specifically, we relax and tighten the inactivity window to create two alternative datasets with different imbalance ratios. The relaxed dataset defines churners based on a 4-day consecutive inactivity window initiated within the first 4 days, which produces a slightly less imbalanced sample of 61 churned and 1,119 non-churned players, while the tightened dataset enforces a 10-day inactivity threshold within the first 10 days, thereby generating a more severely skewed distribution of 25 churned and 1,125 non-churned players.

The prediction results for these two alternative datasets are presented in Table 12 (corresponding to the 4-day threshold) and Table 13 (corresponding to the 10-day threshold). In line with our primary findings, the proposed MSI framework consistently and significantly outperforms all baseline models (BSM, WN1M, and WN2M) and single social influence models (FRDM and PVEM) across all four classification algorithms. For instance, even under the severely imbalanced 10-day threshold dataset (Table 13), MSIM utilizing the SVM (RBF kernel) maintains exceptional performance, achieving an accuracy of 0.9574, an F1-score of 0.9594, and an outstanding AUC of 0.9897.

**Table 12:** Prediction results of four classification algorithms for each model under 4-day threshold dataset.

Classification Algorithm	Model	Accuracy	Precision	Recall	F1-score	AUC
LR	BSM	0.7510	0.7217	0.8099	0.7630	0.8238
	FRDM	**0.7882**	**0.7578**	0.8437	**0.7980**	0.8465
	PVEM	0.7741	0.7537	0.8149	0.7829	0.8419
	WN1M	0.7402	0.7157	0.7898	0.7506	0.8129
	MSIM	0.7854	0.7486	**0.8519**	0.7968	**0.8522**
	WN2M	0.7308	0.7151	0.7719	0.7421	0.8031
SVM (linear kernel)	BSM	0.7567	0.7102	0.8625	0.7787	0.8261
	FRDM	0.7859	**0.7414**	0.8858	0.8069	0.8391
	PVEM	0.7746	0.7314	0.8709	0.7945	0.8391
	WN1M	0.7448	0.7035	0.8369	0.7643	0.8098
	MSIM	**0.7892**	0.7381	**0.9018**	**0.8115**	**0.8471**
	WN2M	0.7444	0.7092	0.8266	0.7627	0.8152
SVM (RBF kernel)	BSM	0.7846	0.7367	0.8778	0.8005	0.8772
	FRDM	0.8461	0.8078	0.9089	0.8552	0.9298
	PVEM	0.8239	0.7791	0.9040	0.8365	0.9135
	WN1M	0.6181	0.6113	0.6391	0.6240	0.6661
	MSIM	**0.8600**	**0.8251**	**0.9180**	**0.8686**	**0.9413**
	WN2M	0.5806	0.5709	0.6056	0.5873	0.6170
GNB	BSM	0.6891	0.6331	0.9031	0.7443	0.8110
	FRDM	0.7054	0.6466	**0.9096**	0.7557	0.8370
	PVEM	0.6900	0.6362	0.8729	0.7359	0.8043
	WN1M	0.6951	0.6401	0.9041	0.7494	0.8224
	MSIM	**0.7171**	**0.6593**	0.9002	**0.7610**	**0.8430**
	WN2M	0.6965	0.6425	0.9080	0.7521	0.8161

**Table 13:** Prediction results of four classification algorithms for each model under 10-day threshold dataset.

Classification Algorithm	Model	Accuracy	Precision	Recall	F1-score	AUC
LR	BSM	0.8755	0.8439	0.9205	0.8804	0.9332
	FRDM	0.9142	0.8812	0.9568	0.9173	0.9552
	PVEM	0.8793	0.8543	0.9130	0.8825	0.9454
	WN1M	0.8525	0.8273	0.8864	0.8555	0.9243
	MSIM	**0.9359**	**0.8901**	**0.9935**	**0.9389**	**0.9647**
	WN2M	0.8540	0.8425	0.8745	0.8579	0.9254
SVM (linear kernel)	BSM	0.8737	0.8236	0.9516	0.8828	0.9392
	FRDM	0.9226	0.8811	0.9788	0.9272	0.9533
	PVEM	0.8936	0.8443	0.9686	0.9020	0.9416
	WN1M	0.8729	0.8287	0.9407	0.8809	0.9371
	MSIM	**0.9427**	**0.8979**	**1.0000**	**0.9462**	**0.9695**
	WN2M	0.8690	0.8320	0.9269	0.8766	0.9347
SVM (RBF kernel)	BSM	0.8870	0.8428	0.9527	0.8942	0.9548
	FRDM	0.9465	0.9059	0.9968	0.9492	0.9809
	PVEM	0.9171	0.8642	0.9886	0.9220	0.9697
	WN1M	0.7791	0.7745	0.7827	0.7782	0.8665
	MSIM	**0.9574**	**0.9238**	**0.9979**	**0.9594**	**0.9897**
	WN2M	0.7479	0.7521	0.7442	0.7475	0.8310
GNB	BSM	0.7584	0.6743	0.9996	0.8052	0.8836
	FRDM	0.7867	0.7016	0.9998	0.8245	**0.9387**
	PVEM	0.7733	0.6868	1.0000	0.8142	0.8973
	WN1M	0.7690	0.6808	0.9998	0.8100	0.8963
	MSIM	**0.8054**	**0.7213**	**1.0000**	**0.8380**	0.9373
	WN2M	0.7747	0.6892	1.0000	0.8160	0.8981

Crucially, in highly imbalanced datasets, accuracy alone can be misleading; however, the MSI framework demonstrates robust improvements in precision, recall, and F1-score across both datasets. This indicates that our model does not merely predict the majority class but possesses a genuine capability to identify the minority churn instances based on complex social structural features. The stability of these metrics across varying churn definitions and imbalance ratios strongly validates the robustness, generalizability, and practical reliability of our proposed framework.

### 4.9 Sensitivity analysis

**Fig 6:**
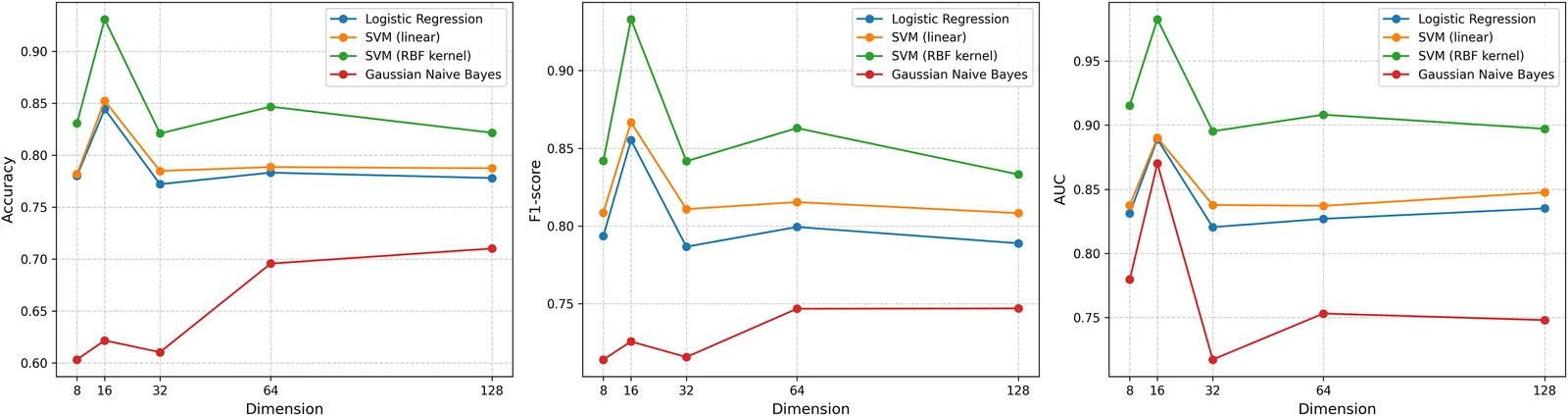
Sensitivity analysis results.

To rigorously analyze the impact of the embedding dimension on our proposed MSI-based Player Churn Prediction framework, we conducted a parameter sensitivity study by varying the embedding dimension size d∈{8,16,32,64,128}. The performance variations across the four classification algorithms are visually illustrated in Fig 6. The experimental results reveal a highly consistent, non-monotonic trend across the models, most notably in LR and SVM (both linear and RBF kernels). Specifically, as the embedding dimension increases from d=8 to d=16, there is a sharp and significant surge in all predictive metrics. The framework reaches its absolute peak performance at d=16. For instance, under the SVM (RBF kernel) classifier, MSIM achieves its highest accuracy of 0.9307, F1-score of 0.9330, and an outstanding AUC of 0.9826. However, as the dimension size continues to increase beyond 16 (i.e., d=32,64,128), the performance experiences a notable drop and subsequently plateaus at a suboptimal level. This phenomenon can be theoretically attributed to the intrinsic structural complexity of the gaming networks. At d=8, the low-dimensional space creates an information bottleneck, leading to under-fitting, where the model lacks the capacity to encapsulate the highly complex, multi-scale hierarchical structures of the player relationships. Conversely, at d≥32, the model suffers from the curse of dimensionality and over-fitting. The excessively high-dimensional embeddings begin to capture noise, sparsity artifacts, and redundant structural details rather than meaningful social patterns, thereby degrading the downstream classification performance.

In conclusion, our empirical evaluation explicitly identifies d=16 as the optimal embedding dimension for our dataset. It provides the optimal balance by possessing sufficient capacity to encode the synergistic multiplex social influence while remaining compact enough to filter out topological noise.

## 5 Conclusions

This study proposes a novel player churn prediction framework driven by Multiplex Social Influence. By explicitly modeling the distinct yet overlapping interactions between players—specifically, explicit friendship ties and implicit PvE teammate relationships—the framework constructs multiplex social networks to capture a holistic view of player dynamics. To efficiently extract these features, we employ the MLNRL algorithm, which preserves complex, multi-scale structural information while ensuring high computational scalability. These relational embeddings are subsequently concatenated and integrated with traditional individual behavioral attributes. Through rigorous ablation studies and extensive robustness checks—including evaluations under severe data imbalance and comparisons with alternative graph embedding algorithms (e.g., GCN, GraphSAGE)—we definitively demonstrate that integrating multiplex relationship information yields superior and highly stable prediction performance. The results underscore both the effectiveness and the critical necessity of aggregating heterogeneous social structures to capture the synergistic effects driving player churn.

Furthermore, this study makes three critical theoretical and methodological contributions. First, it advances existing user behavior research by conceptualizing a novel prediction paradigm grounded in multiplex social influence. We theoretically and empirically prove that heterogeneous social ties capture orthogonal behavioral signals, and their integration generates a powerful synergistic effect, significantly mitigating the observational biases inherent in single-network models. Second, from a methodological perspective, this study validates the superiority of deep adversarial network embeddings for modeling massive social graphs. By implicitly factorizing the SPPMI matrix and utilizing a Laplacian cascade, our approach efficiently preserves hierarchical structures while overcoming the over-smoothing problem common in standard graph neural networks, offering a highly scalable solution for social network analysis. Finally, our findings provide robust theoretical and practical support for domains extending beyond online gaming. The framework’s ability to maintain high precision and recall under extreme class imbalance offers highly actionable value for operational retention management. Moreover, the demonstrated multiplex architecture can be readily generalized to other complex network environments, such as customer churn in telecommunications and user behavior mining in online retail contexts.

This research also offers profound managerial implications for industry practitioners. First, at the operational level, the proposed MSI-based Player Churn Prediction framework achieves superior prediction performance—particularly demonstrating high precision and recall under severely imbalanced real-world conditions. This equips game operators with a highly reliable tool to accurately identify high-risk churners. Consequently, companies can optimize their operational resource allocation by proactively implementing targeted retention interventions (e.g., personalized incentives or cross-promotion of related titles) for valuable players, while avoiding the squandering of marketing budgets on false positives. For players exhibiting an inevitable trajectory toward churn, managers can strategically pivot to alternative monetization tactics, such as serving third-party in-game advertisements, thereby mitigating potential revenue attrition. Second, from a strategic perspective, the validation of multiplex social influence highlights the necessity of cultivating and monitoring diverse user interactions. Game developers should incentivize not only the formation of explicit friendships but also the engagement in implicit, task-oriented collaborations (e.g., PvE events), as the synergy of these multiplex relationships significantly bolsters player stickiness. Finally, the practical utility of this framework extends far beyond the gaming industry. Managers in highly competitive sectors characterized by rich user interactions—such as telecommunications, decentralized finance, and social e-commerce—can seamlessly integrate our multiplex network embedding approach into their CRM (Customer Relationship Management) systems. By holistically capturing the multiplex social influences acting upon their customers, firms can achieve more granular user profiling, anticipate attrition with greater precision, and design highly effective, data-driven intervention strategies, ultimately maximizing long-term customer lifetime value.

Despite its contributions, this study has several limitations that suggest avenues for future research. First, as the primary objective of this study is to demonstrate the effectiveness of multiplex social influence in improving player churn prediction performance, supervised machine learning models are employed for comparative evaluation. However, from a managerial perspective, predicting the timing of player churn is often of equal importance. Future studies may therefore adopt survival analysis or time-to-event modeling techniques to estimate when the churn is likely to occur. Second, while our framework effectively demonstrates the superiority of multiplex social networks, the algorithmic integration of these networks could be further deepened. Currently, the framework relies on late-stage concatenation, which implies that the MLNRL algorithm learns the embeddings of the friendship and PvE teammate networks independently. Consequently, there is a lack of direct cross-network information flow or interaction during the embedding training phase. Inspired by recent advancements in graph representation learning, future research should explore more sophisticated integration strategies, such as developing a joint optimization framework. By employing unified multiplex graph neural networks that simultaneously learn and share parameters across multiple network layers in an end-to-end manner, models could capture the synergistic interactions between different social ties more fundamentally. Such advanced architectures are highly promising for further enhancing the representation of multiplex social influence and pushing the boundaries of prediction performance.

## Supporting information

S1 File Total data anonymized. (CSV)
